# Supplementary figures and images for: Mathematical modeling of the Candida albicans yeast to hyphal transition reveals novel control strategies
Source: PLoS Comput Biol. 2021 Mar 29;17(3):e1008690. doi: 10.1371/journal.pcbi.1008690 (PMC8031856; doi:10.1371/journal.pcbi.1008690)

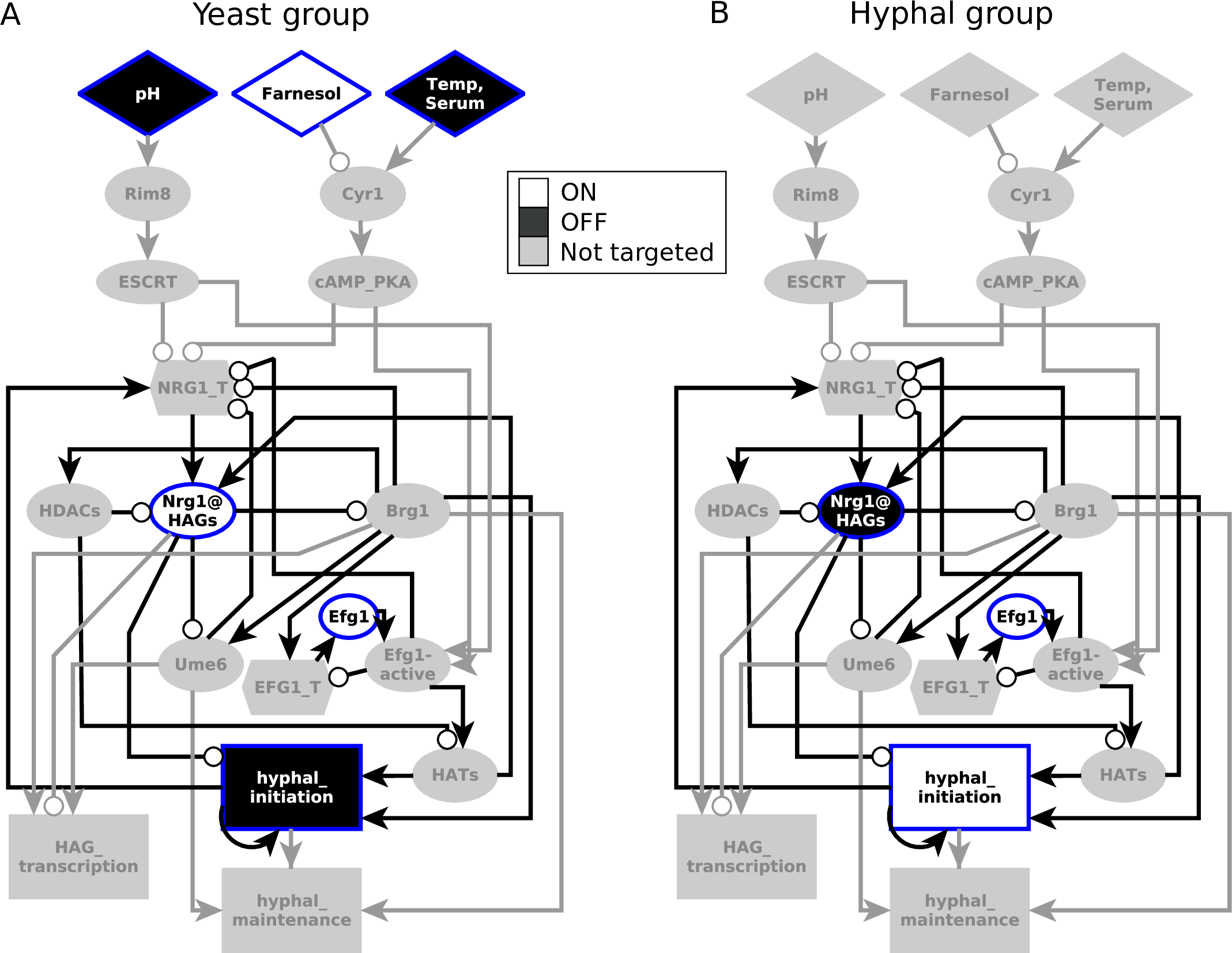

Supplement: S1 Fig — FVS control strategies to drive the system into a target attractor (group). Bold edges participate in feedback loops that are broken by controlling the values of the FVS. Nodes and edges that are irrelevant to feedback vertex control are shown in light grey, while nodes of the FVS are shown with a blue outline, and the nodes are colored based on the values they require for FVS control. (A) FVS control predicts fixing environmental conditions as pH = 0 and either Farnesol = 1 or Temperature = 0, and then fixing Nrg1@HAGs = Efg1 = 1 and hyphal_initiation = 0 will force the system into the yeast attractor. Instead of Efg1, EFG1_T or Efg1_active would also be suitable targets. (B) FVS control predicts fixing Nrg1@HAGs = 0, and Efg1 = hyphal_initiation = 1 will force the system into a hyphal attractor. (TIF) [file pcbi.1008690.s001.tif]

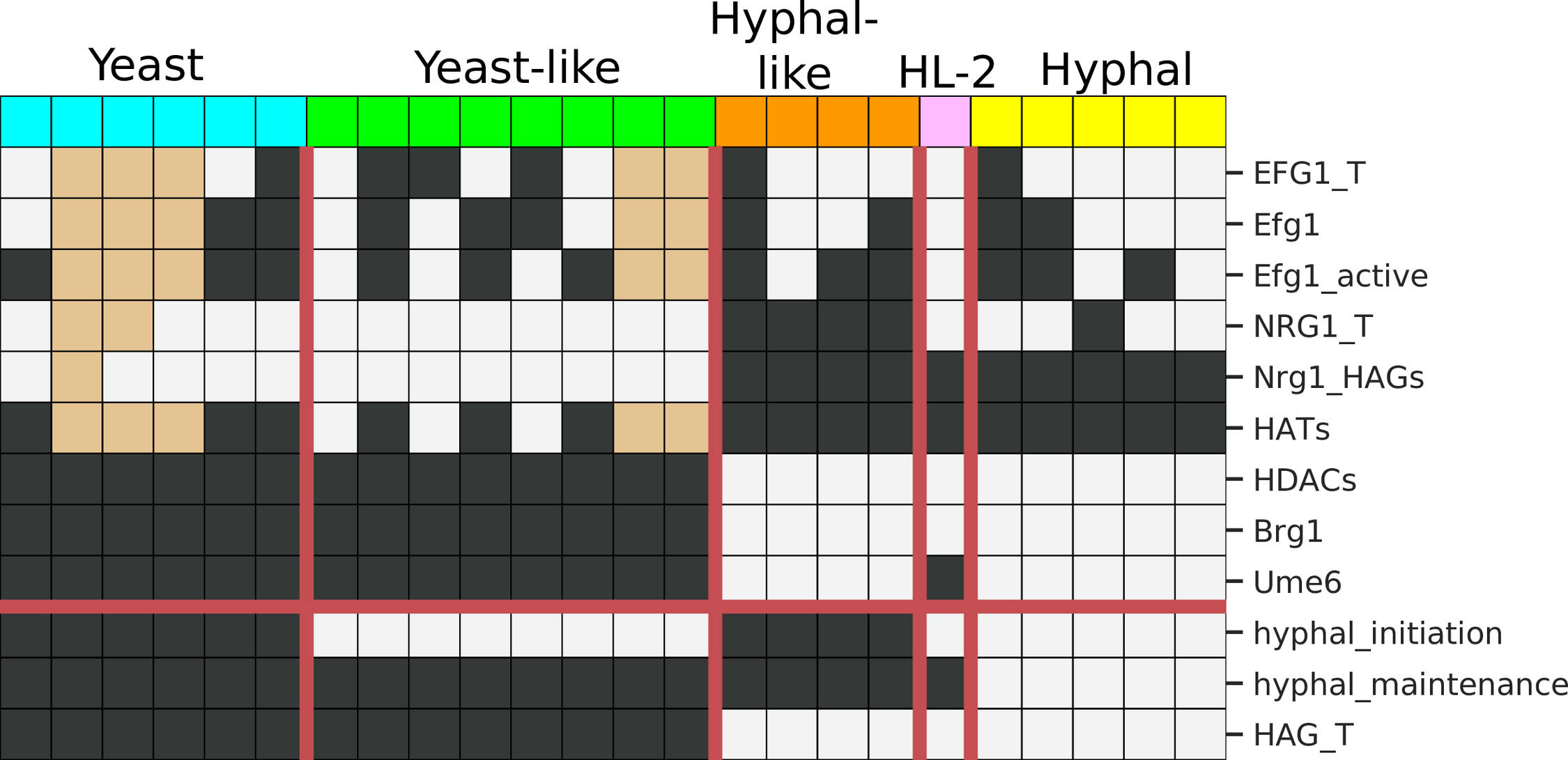

Supplement: S2 Fig — Single-node deletions or activations lead to new attractors. All attractors reached under YHT inducing conditions (pH = 1, Temperature = Farnesol = 0) are shown. Phenotype classification was performed as before, except the values of environment source nodes (pH, Farnesol, and Temperature), signaling intermediaries (Rim8, ESCRT, Cyr1, cAMP/PKA), and the individual perturbed node are ignored. One attractor emerged which did not fit the phenotype classifications defined previously, which we here call hyphal-like 2 (HL2). It corresponds to deletion of UME6, and has hyphal_initiation = 1, HAG_transcription = 1, and hyphal_maintenance = 0. (TIF) [file pcbi.1008690.s002.tif]
