# Supplementary material for: Mathematical modeling of the Candida albicans yeast to hyphal transition reveals novel control strategies
Source: PLoS Comput Biol. 2021 Mar 29;17(3):e1008690. doi: 10.1371/journal.pcbi.1008690 (PMC8031856; doi:10.1371/journal.pcbi.1008690)
Supplement: S1 Table — Compilation of published experimental intervention results and comparison with the relevant model results. The first column describes the intervention. The second column indicates the environmental condition used in the experiments. The composition of the various media is the following: YPD (also denoted YEPD) medium: yeast extract-peptone dextrose, pH = 7 at the beginning of culture (it decreases as the yeast breaks down dextrose); B-medium: 0.67% yeast nitrogen base, 2% Na-succinate, pH = 6.5; RPMI-1640 supplemented with L-glutamine and buffered with morpholinepropanesulfonic acid (MOPS), pH = 7.0; Spider medium: nutrient broth, mannitol, K2PO4, agar, pH = 7.2. Because all of these experiments start with a dilution of cells into fresh medium, the farnesol level is expected to be very low (equivalent with Farnesol = 0 in the model). The experimental conditions that lead to successful YHT in wildtype cells are shown in blue font; the rest are expected to be yeast-favoring environments. The third column summarizes the experimental result and the fifth column indicates the reference. The fourth column indicates the attractor repertoire of the model in the simulated intervention and environmental condition closest to the experiment. The model results that deviate from experimental observations are shown in red font. The rest of the model results are consistent with experimental observations. (DOCX) [file pcbi.1008690.s003.docx]

**S1 Table. Model validation by comparison with literature.**

| **Expt** | **Environment** | **Phenotype(s)** | **Prediction** | **Ref(s)** |
| --- | --- | --- | --- | --- |
| WT | YPD at 30°C | Yeast-form | 100% Y | [1] |
|  | YPD + 10% serum at 37°C | Extensive hyphal growth | 37% H, 51% HL, 12% YL | [1] |
| Efg1 = 0 | RPMI 1640 medium at 37°C | Deletion of *EFG1* leads to inability to filament, no biofilms, sparse monolayers of loosely attached elongated cells. | 100% Y | [2] |
|  |  |  |  |  |
|  | Spider medium at 37°C | Deletion of *EFG1* leads to inability to form biofilms and hyphae. |  | [3] |
| Efg1_active = 1 | B-Medium | Pseudohyphae | 100% Y | [4] |
|  | B-Medium +5% horse serum | Hyphae | 53% H, 27% HL, 20% YL | [4] |
| NRG1_T = 0 | YPD at 25°C | Pseudohyphae, HAGs are expressed. | 100% HL | [5] |
|  | YPD at 30°C | Hyphae |  | [6] |
|  | YPD + 20% serum at 37°C | Hyphae | 22% H, 78% HL | [5] |
| NRG1_T = 1 | Multiple hyphal-growth-  inducing liquid media at 37°C | Inhibits hyphal growth in all conditions. | 100% Y | [7,8] |
| Brg1 = 0 | YPD + 10% serum at 37°C | Competent germ tube formation, defective hyphal elongation. | 100% Y | [9] |
| Brg1 = 1 | YEPMaltose medium at 25° C | Cells remained in yeast-form. | 100% HL | [9] |
|  | YEPMaltose medium at 37° C | Ectopic expression of *BRG1* sustains hyphal growth. | 3% H, 97% HL | [9] |
| HATs = 0 | YPD at 35°C | Deletion of *ESA1*, encoding a subunit of the HAT NuA4 complex blocked hyphal initiation. | 100% Y | [10] |
|  | YPD + 10% bovine serum at 37°C | Deletion of *YNG2*, encoding an active subunit of NuA4, diminished HAG transcription and formed few filaments. | 100% HL | [11] |
| HDACs = 0 | YPD + 10% serum at 37°C | Defective in sustained hyphal growth, Nrg1@HAGs increases after hyphal initiation. | 100% YL | [1] |
| Ume6 = 0 | YEPD + 10% fetal calf serum at 37 °C | Deletion of *UME6* leads to strong reduction in HAG transcription and significantly shorter filaments. | 42% HL2, 41% HL, 17% YL | [12] |
|  | YPD + 10% fetal bovine serum at 37 °C | Double deletion of *SSN6* and *RDP31* leads to decreased Ume6, and impaired filament extensions |  | [13] |
|  | YEPD at 30 °C | Yeast | 100% Y | [12] |
| Ume6=1 | YEPD at 30 °C | High level, constitutive expression of *UME6* leads to hyphal formation. Intermediate-level constitutive expression of *UME6* leads to a mixture of hyphae and pseudohyphae. | 100% HL | [14] |
| Delete edge from HDACs to HATs | YPD with 10% serum at 37°C | A constitutively acetylated Yng2 (active subunit of NuA4) could not sustain HAG transcription and hyphal elongation. | 100% YL | [1] |
| Rim8 = 1 or ESCRT =1 | Medium 199 at pH=4 and 29 °C | High Rim101 activity induced hyphal growth even at pH and temperature favoring the yeast-form. | 37% H, 51% HL, 12% YL | [15] |

**Reference**

1. Lu Y, Su C, Wang A, Liu H. Hyphal development in Candida albicans requires two temporally linked changes in promoter chromatin for initiation and maintenance. PLoS Biol. 2011;9: e1001105.

2. Ramage G, VandeWalle K, López-Ribot JL, Wickes BL. The filamentation pathway controlled by the Efg1 regulator protein is required for normal biofilm formation and development in Candida albicans. FEMS Microbiol Lett. 2002;214: 95–100.

3. Nobile CJ, Fox EP, Nett JE, Sorrells TR, Mitrovich QM, Hernday AD, et al. A recently evolved transcriptional network controls biofilm development in Candida albicans. Cell. 2012;148: 126–138.

4. Stoldt VR, Sonneborn A, Leuker CE, Ernst JF. Efg1p, an essential regulator of morphogenesis of the human pathogen Candida albicans, is a member of a conserved class of bHLH proteins regulating morphogenetic processes in fungi. EMBO J. 1997;16: 1982–1991.

5. Murad AM, Leng P, Straffon M, Wishart J, Macaskill S, MacCallum D, et al. NRG1 represses yeast-hypha morphogenesis and hypha-specific gene expression in Candida albicans. EMBO J. 2001;20: 4742–4752.

6. Braun BR. NRG1, a repressor of filamentous growth in C.albicans, is down-regulated during filament induction. The EMBO Journal. 2001. pp. 4753–4761. doi:10.1093/emboj/20.17.4753

7. Park Y-N, Morschhäuser J. Tetracycline-inducible gene expression and gene deletion in Candida albicans. Eukaryot Cell. 2005;4: 1328–1342.

8. Saville SP, Lazzell AL, Bryant AP, Fretzen A, Monreal A, Solberg EO, et al. Inhibition of filamentation can be used to treat disseminated candidiasis. Antimicrob Agents Chemother. 2006;50: 3312–3316.

9. Lu Y, Su C, Liu H. A GATA Transcription Factor Recruits Hda1 in Response to Reduced Tor1 Signaling to Establish a Hyphal Chromatin State in Candida albicans. PLoS Pathogens. 2012. p. e1002663. doi:10.1371/journal.ppat.1002663

10. Wang X, Zhu W, Chang P, Wu H, Liu H, Chen J. Merge and separation of NuA4 and SWR1 complexes control cell fate plasticity in Candida albicans. Cell Discovery. 2018. doi:10.1038/s41421-018-0043-0

11. Lu Y, Su C, Mao X, Raniga PP, Liu H, Chen J. Efg1-mediated recruitment of NuA4 to promoters is required for hypha-specific Swi/Snf binding and activation in Candida albicans. Mol Biol Cell. 2008;19: 4260–4272.

12. Banerjee M, Thompson DS, Lazzell A, Carlisle PL, Pierce C, Monteagudo C, et al. UME6, a novel filament-specific regulator of Candida albicans hyphal extension and virulence. Mol Biol Cell. 2008;19: 1354–1365.

13. Lee J-E, Oh J-H, Ku M, Kim J, Lee J-S, Kang S-O. Ssn6 has dual roles in Candida albicans filament development through the interaction with Rpd31. FEBS Lett. 2015;589: 513–520.

14. Carlisle PL, Banerjee M, Lazzell A, Monteagudo C, López-Ribot JL, Kadosh D. Expression levels of a filament-specific transcriptional regulator are sufficient to determine Candida albicans morphology and virulence. Proc Natl Acad Sci U S A. 2009;106: 599–604.

15. Barkani AE, El Barkani A, Kurzai O, Fonzi WA, Ramon A, Porta A, et al. Dominant Active Alleles of RIM101(PRR2) Bypass the pH Restriction on Filamentation of Candida albicans. Molecular and Cellular Biology. 2000. pp. 4635–4647. doi:10.1128/mcb.20.13.4635-4647.2000
