# Supplementary material for: Mathematical modeling of the Candida albicans yeast to hyphal transition reveals novel control strategies
Source: PLoS Comput Biol. 2021 Mar 29;17(3):e1008690. doi: 10.1371/journal.pcbi.1008690 (PMC8031856; doi:10.1371/journal.pcbi.1008690)
Supplement: S1 Text — Justification for the choice of regulatory functions, along with relevant citations. (DOCX) [file pcbi.1008690.s004.docx]

**Text S1. Explanation of the regulatory functions of the model.**

**pH* = pH**

**Rim8* = pH**

**ESCRT* = Rim8**

The pathway of sensing and responding to the environmental pH level is based on the Rim family of proteins. Several transmembrane proteins connected to intracellular Rim8 sense environmental pH. Neutral or alkaline pH (pH > 6) causes a signaling cascade through Rim8 that involves the endosomal sorting complex required for transport (ESCRT) and leads to cleavage of the transcription factor Rim101 [1]. To simplify we only include two elements, Rim8 and ESCRT, to describe activation of the full pathway in response to neutral/alkaline pH, which is abstracted to the Boolean state pH = 1.

**Temperature* = Temperature**

**Serum* = Serum**

**Farnesol* = Farnesol**

**Cyr1* = Temperature and Serum and not Farnesol**

**cAMP_PKA* = Cyr1**

Cyclic AMP (cAMP) production by the adenylate cyclase Cyr1 and the subsequent activation of protein kinase A (PKA) is a major hyphal growth inducing pathway [2]. Serum promotes filamentation through the muramyl peptide, which binds to Cyr1 and stimulates its adenylate cyclase activity [3]. Incubation at 37 C leads to the activation of Cyr mediated by the molecular chaperone Hsp90 [4]. The quorum sensing molecule farnesol inhibits the activity of Cyr1 [5]. As the environments that consistently yield YHT include serum, have a temperature of 37 °C and a low cell density [6], we assume that Cyr1 is activated only if all three conditions are simultaneously satisfied. In the analysis of the model we merge the effects of serum and temperature into a single node, which we call “Temperature”.

**Efg1_T* = Brg1 or not Efg1_active**

**Efg1* = Efg1_T**

**Efg1_active* = (ESCRT or cAMP_PKA) and Efg1**

The Efg1 protein downregulates *EFG1* transcription in a negative self-regulation loop [7]. Brg1 can bind to the Efg1 promoter, it is unclear if it causes an expression change [8,9]. We include a positive effect from Brg1 to *EFG1* transcription. (This effect does not seem necessary for most findings.) We assume that either Brg1 or the absence of Efg1_active can maintain Efg1 transcription. We separate out the active form of the Efg1 protein (Efg1_active) from the generic Efg1 protein, which is translated from the *EFG1* transcript (Efg1_T). Efg1_active is induced in response to cAMP_PKA [10]. Based on the observation that the filamentation-inducing effect of constitutive activation of Rim101 is abolished by knocking out *EFG1* [1], we assume that the Rim pathway (ESCRT in the model) also leads to the activation of Efg1 (*i.e.* to Efg1_active).

This set of functions reproduces the observation that there is a reduction of Efg1 expression in response to YHT inducing stimuli [11]. These functions predict that Efg1 expression will be restored after Brg1 turns on.

**NRG1_T* = not Brg1 and not Ume6 and not (Efg1_active and (ESCRT or cAMP_PKA)) or hyphal_initiation**

Equivalently,

**NRG1_T* = not {Brg1 or Ume6 or [Efg1_active and (ESCRT or cAMP_PKA)]} or hyphal_initiation**

Signals that induce cAMP/PKA lead to the downregulation of *NRG1* [12]. We assume that pH acts similarly to inducers of cAMP/PKA in terms of how they affect the core network. Despite the downregulation of Efg1 expression following signals, Efg1 is required for downregulation of Nrg1 [13]. We implement this by considering that the active Efg1 is required, in collaboration with cAMP/PKA or pH signaling, for the downregulation of Nrg1.

Brg1 negatively influences the stability of the *NRG1* transcript by upregulating an antisense transcript [14]. Ume6 overexpression can repress Nrg1, and *UME6* KO reduces the downregulation of Nrg1 [15]. We include Ume6 as a sufficient inhibitor of NRG1_T.

We do not know the mechanism through which *NRG1* expression is restored following hyphal initiation, so we include a positive edge from hyphal_initiation to Nrg1_T. A possibility to consider in the future is that this effect is through Brg1, but time courses seem to suggest Brg1 increase and Nrg1 decrease are simultaneous [13].

**Nrg1@HAGs* = NRG1_T and (not HDACs or HATs)**

Equivalently,

**Nrg1@HAGs* = NRG1_T and not (HDACs and not HATs)**

The binding of Nrg1 to the promoter region of HAGs requires the expression of the NRG1 transcript and protein, and the correct chromatin state. HDACs (such as Hda1) deacetylate histones at the Nrg1 binding site of HAGs, preventing Nrg1 from binding at HAGs. However, the effect of HDACs is dependent on Hda1 first deacetylating the Yng2 subunit of NuA4 (a main contributor to the node HATs), leading to HATs degradation. After a YHT inducing signal, Nrg1@HAGs goes down because of Nrg1 expression downregulation. Even though Nrg1 expression can go back up, Nrg1@HAGs does not because of HDACs, which block Nrg1 from binding to the promoters of HAGs. If HATs are not degraded by HDACs, Nrg1 can return to its binding site [12,13].

**Brg1* = not Nrg1@HAGs**

*BRG1* is one of the HAGs whose transcription Nrg1 blocks. Nrg1 and Brg1 form a mutual inhibitory loop [14]. Efg1, several other transcription factors encoded by HAGs, and Brg1 itself bind the promoter region of *BRG1* [8]. As the strength of these effects is not known, we make Nrg1@HAGs the sole regulator of Brg1.

**HDACs* = Brg1**

Brg1 recruits the HDAC Hda1 to the promoters of HAGs [12].

**HATs* = Efg1_active and not HDACs**

HATs (such as NuA4) are induced by Efg1 [16]. The Yng2 subunit of NuA4 is deacetylated by Hda1, which causes its degradation [13]. We represent this effect as a negative regulation between HDACs and HATs.

**Ume6* = Brg1 and not Nrg1@HAGs**

Similarly to other HAGs, *UME6* transcription is regulated by Brg1 and HDACs [12]. We assume the effect of HDACs is through Nrg1@HAGs downregulation. Since HDAC KO disrupts *UME6* transcription [12], we assume an AND rule between Brg1 and "not Nrg1@HAGs"

**hyphal_initiation* = (HATs and Brg1 and not Nrg1@HAGs) or hyphal_initiation**

The recruitment of the NuA4 complex to the promoters of HAGs is required for nucleosomal H4 acetylation at the promoters during hyphal induction [16]. We implement this by assuming that HATs are required for hyphal initiation. We also assume that inactive Nrg1@HAGs and active Brg1 are necessary for hyphal initiation.

We assume that hyphal initiation is irreversible.

**HAG_transcription* = (Brg1 or Ume6) and not Nrg1@HAGs**

This phenotypic outcome node represents the process of transcription of hyphal-associated genes. Although its meaning is partially overlapping with the meaning of the process node “hyphal_initiation”, in our model it is possible to achieve HAG transcription while bypassing the standard mechanisms of hyphal initiation, by directly activating core TF drivers of the hyphal program. Having such a node allows the incorporation of the observation that Ume6 overexpression in yeast-favoring environments leads to transcription of HAGs and formation of pseudohyphae or hyphae [17]. We assume that the activity of either Brg1 or Ume6 and the inactivity of Nrg1@HAGs is needed for HAG transcription.

**hyphal_maintenance* = (Ume6 and not Nrg1@HAGs) and hyphal_initiation**

The phenotypic outcome node hyphal_maintenance expresses the hyphal development stage that follows hyphal initiation. We assume that hyphal initiation needs to have been completed for this phase to commence. The transcription factor Ume6 is expressed during hyphal elongation, and controls the level and duration of hyphal-specific genes and is important for hyphal elongation [13]. *UME6* levels are sufficient (even after HDA1 KO) for hyphal-maintenance [12,13]. Since Brg1 and HDAcs regulate *UME6* expression, we assume the effect of Brg1 and HDACs on hyphal maintenance is through *UME6*.

**Reference**

1. Barkani AE, El Barkani A, Kurzai O, Fonzi WA, Ramon A, Porta A, et al. Dominant Active Alleles of RIM101(PRR2) Bypass the pH Restriction on Filamentation of Candida albicans. Molecular and Cellular Biology. 2000. pp. 4635–4647. doi:10.1128/mcb.20.13.4635-4647.2000

2. Biswas S, Van Dijck P, Datta A. Environmental sensing and signal transduction pathways regulating morphopathogenic determinants of Candida albicans. Microbiol Mol Biol Rev. 2007;71: 348–376.

3. Xu X-L, Lee RTH, Fang H-M, Wang Y-M, Li R, Zou H, et al. Bacterial peptidoglycan triggers Candida albicans hyphal growth by directly activating the adenylyl cyclase Cyr1p. Cell Host Microbe. 2008;4: 28–39.

4. Shapiro RS, Uppuluri P, Zaas AK, Collins C, Senn H, Perfect JR, et al. Hsp90 orchestrates temperature-dependent Candida albicans morphogenesis via Ras1-PKA signaling. Curr Biol. 2009;19: 621–629.

5. Hall RA, Turner KJ, Chaloupka J, Cottier F, De Sordi L, Sanglard D, et al. The quorum-sensing molecules farnesol/homoserine lactone and dodecanol operate via distinct modes of action in Candida albicans. Eukaryot Cell. 2011;10: 1034–1042.

6. Sudbery P, Gow N, Berman J. The distinct morphogenic states of Candida albicans. Trends Microbiol. 2004;12: 317–324.

7. Tebarth B, Doedt T, Krishnamurthy S, Weide M, Monterola F, Dominguez A, et al. Adaptation of the Efg1p morphogenetic pathway in Candida albicans by negative autoregulation and PKA-dependent repression of the EFG1 gene. J Mol Biol. 2003;329: 949–962.

8. Nobile CJ, Fox EP, Nett JE, Sorrells TR, Mitrovich QM, Hernday AD, et al. A recently evolved transcriptional network controls biofilm development in Candida albicans. Cell. 2012;148: 126–138.

9. Hnisz D, Bardet AF, Nobile CJ, Petryshyn A, Glaser W, Schöck U, et al. A histone deacetylase adjusts transcription kinetics at coding sequences during Candida albicans morphogenesis. PLoS Genet. 2012;8: e1003118.

10. Bockmühl DP, Ernst JF. A potential phosphorylation site for an A-type kinase in the Efg1 regulator protein contributes to hyphal morphogenesis of Candida albicans. Genetics. 2001;157: 1523–1530.

11. Stoldt VR, Sonneborn A, Leuker CE, Ernst JF. Efg1p, an essential regulator of morphogenesis of the human pathogen Candida albicans, is a member of a conserved class of bHLH proteins regulating morphogenetic processes in fungi. EMBO J. 1997;16: 1982–1991.

12. Lu Y, Su C, Liu H. A GATA Transcription Factor Recruits Hda1 in Response to Reduced Tor1 Signaling to Establish a Hyphal Chromatin State in Candida albicans. PLoS Pathogens. 2012. p. e1002663. doi:10.1371/journal.ppat.1002663

13. Lu Y, Su C, Wang A, Liu H. Hyphal development in Candida albicans requires two temporally linked changes in promoter chromatin for initiation and maintenance. PLoS Biol. 2011;9: e1001105.

14. Cleary IA, Lazzell AL, Monteagudo C, Thomas DP, Saville SP. BRG1 and NRG1 form a novel feedback circuit regulating Candida albicans hypha formation and virulence. Molecular Microbiology. 2012. pp. 557–573. doi:10.1111/j.1365-2958.2012.08127.x

15. Banerjee M, Thompson DS, Lazzell A, Carlisle PL, Pierce C, Monteagudo C, et al. UME6, a novel filament-specific regulator of Candida albicans hyphal extension and virulence. Mol Biol Cell. 2008;19: 1354–1365.

16. Lu Y, Su C, Mao X, Raniga PP, Liu H, Chen J. Efg1-mediated recruitment of NuA4 to promoters is required for hypha-specific Swi/Snf binding and activation in Candida albicans. Mol Biol Cell. 2008;19: 4260–4272.

17. Carlisle PL, Banerjee M, Lazzell A, Monteagudo C, López-Ribot JL, Kadosh D. Expression levels of a filament-specific transcriptional regulator are sufficient to determine Candida albicans morphology and virulence. Proc Natl Acad Sci U S A. 2009;106: 599–604.
